# Trends in Disenrollment and Reenrollment Within US Commercial Health Insurance Plans, 2006-2018

**DOI:** 10.1001/jamanetworkopen.2022.0320

**Published:** 2022-02-24

**Authors:** Hanming Fang, Molly Frean, Gosia Sylwestrzak, Benjamin Ukert

**Affiliations:** 1 Department of Economics, University of Pennsylvania, Philadelphia; 2 Analysis Group, Boston, Massachusetts; 3 Anthem Inc, Wilmington, Delaware; 4 Department of Health Policy and Management, School of Public Health, Texas A&M University, College Station

## Abstract

**Importance:**

The commercial health insurance market is characterized by consistently high enrollee turnover. Turnover can disrupt care continuity for patients and create challenges for insurers in managing the health of their enrollee populations. Yet the extent to which enrollees reenroll is not widely known.

**Objective:**

To characterize rates of disenrollment (hereafter, *external turnover*) and reenrollment in commercial health plans.

**Design, Setting, and Participants:**

In this retrospective longitudinal cohort study, trends in turnover and reenrollment in commercial health plans between January 1, 2006, and August 31, 2018, were analyzed. Data analysis was conducted from January 21, 2020, through December 23, 2021. Participants included 3 018 633 primary members and their dependents with employer-sponsored coverage.

**Main Outcomes and Measures:**

Primary outcomes included external turnover from commercial coverage and subsequent reenrollment into any line of business with the insurer (commercial, Medicaid Managed Care, and Medicare Advantage). Within commercial coverage, external turnover was analyzed separately for group (ie, employer-sponsored) and individual markets.

**Results:**

In the sample of 3 018 633 members, 50.2% were men; mean (SD) age, including dependents, was 30.68 (19.05) years. A total of 2.2% of members experienced external turnover each month and 21.5% experienced external turnover each year. The individual market had the highest average monthly turnover rate of 3.4% compared with 2.1% in the group market. December had the highest rate of external turnover, with 13.8% experiencing external turnover in the individual market and 6.9% of members experiencing external turnover in the group market. Fourteen percent of the members who left the insurer from an individual plan reenrolled with the insurer after 1 year, and 34% had reenrolled after 5 years. Among members who left the insurer from a group plan, 12% reenrolled after 1 year and 32% reenrolled after 5 years. After 10 years, reenrollment reached 47% in the 2 markets. More than 80% of enrollees returned to the same line of business and within the same state, suggesting findings may generalize to smaller insurers.

**Conclusions and Relevance:**

The findings of this cohort study suggest that insurers may benefit from investing in members’ long-term health outcomes despite substantial short-term turnover rates.

## Key Points

**Question:**

What is the level of disenrollment and reenrollment within commercial health plans?

**Findings:**

In this longitudinal cohort study of 3 018 633 individuals, approximately 1 in 5 members disenrolled from a commercial insurer each year; however, among departing enrollees, approximately 1 in 3 returned to the insurer within 5 years.

**Meaning:**

The findings of this study suggest that insurers can benefit from investing in members’ long-term health outcomes despite substantial short-term turnover rates.

## Teaser

This cohort study examines the rates of turnover in commercial health insurers by members in group and individuals plans.

## Introduction

Unlike other countries with national health systems, the organization of the US health insurance industry creates numerous opportunities for enrollee turnover. In the commercial market (also referred to as the private market), multiple insurers compete for employment-based insurance contracts and individual enrollees. As a result, commercial insurers’ enrollee turnover reflects not only member choices to switch insurers but also member choices to switch employers and employer choices to switch insurers. Medicare and Medicaid are also subject to turnover (churn), with beneficiaries often choosing among privatized plans, and Medicaid enrollees face the added risk of losing eligibility owing to income fluctuations.

Between 15% and 20% of both privately and publicly insured individuals experience coverage disruptions or change plans each year.^1,2,3,4,5,6,7^ Research on health insurance plan choice has identified factors that explain turnover in both contexts, including individual characteristics and behavioral factors, such as inertia or inattention.^8,9,10,11,12,13^ A complementary set of studies has evaluated the direct and relatively short-term consequences of turnover, including how associated disruptions in insurance coverage can lead to disruptions in health care use.^14,15,16^

Turnover, especially within commercial insurance, has implications for the long-term health of insured populations in the US. From a single insurer’s perspective, turnover may occur internally across their menu of plan offerings—often without any gaps in coverage—or externally when a member leaves the insurer entirely. External turnover can be negatively associated with the affordability of insurance when insurers must continuously use resources to attract and enroll new members. In addition, a lack of historical health information on a member at the start of their tenure with an insurer may limit the insurer’s ability to improve health outcomes through tailored care management programs and personalized outreach. Another implication of external turnover, which we highlight in this study, was raised by prior research^17,18,19^: external turnover reduces insurer incentives to invest in preventive care for which benefits accrue over longer time horizons; as such, benefits may not necessarily accrue to the same insurer making the investment. Fang and Gavazza^17^ reported that labor market turnover causes employers to underinvest in their employees’ health during working years, leading to higher health care expenditures during retirement. These authors also noted that specific benefits vary according to industry turnover rates, providing evidence that firms recognize and respond to these dynamic incentives.^18^ Such results explain the findings of Herring,^19^ who documented a negative outcome associated with turnover and actual use of preventive care services. Cebul et al^2^ reported that search frictions in the market for group-based insurance lead to additional turnover beyond that attributable to labor market turnover, further reducing investment incentives.

The objectives of this study were 2-fold. First, we sought to document the extent of external turnover from commercial group and individual plans at one of the country’s largest commercial insurers. Second, we explored the extent to which former enrollees return to the insurer at a later date, including returns to all lines of business (commercial, Medicare Advantage, and Medicaid Managed Care). To our knowledge, no study has explored this possibility empirically.

## Methods

### Data Source

In this cohort study, we used administrative data from Anthem, one of the largest national insurers in the US, from January 1, 2006, through August 31, 2018 (hereafter, *study period*). Data include fully adjudicated medical and pharmacy claims and enrollment data with plan characteristics, such as the line of business and plan type. Anthem operates commercial plans in 14 states, including both employer-sponsored group coverage (fully insured and self-insured) and individual plans (either on or off the Affordable Care Act’s insurance exchanges). In some states, Anthem additionally administers plans through 2 other lines of business: Medicare Advantage and Medicaid Managed Care. The insurer’s predominant plan types in the commercial line of business are preferred provider organizations, health maintenance organizations, and consumer-directed health plans. We used data from a 5% random sample of members enrolled in a commercial plan with the insurer during the study period. For each member, we observed their first segment of commercial coverage during the study period as well as any subsequent enrollment segments in the same or other lines of business. More details on enrollment and sample construction can be found in the eMethods in the Supplement.

This project was determined not to be human participant research by the Texas A&M University Institutional Review Board. This study followed the Strengthening the Reporting of Observational Studies in Epidemiology (STROBE) reporting guideline for cohort studies.

### Outcomes

The primary outcomes we studied were external turnover from commercial coverage and subsequent reenrollment into any line of business with the insurer. We analyzed both outcomes separately for the group and individual markets. We defined external turnover as occurring in a given calendar month if the member was enrolled with the insurer for the full month but no longer enrolled at the start of the following month. External turnover therefore marks the end of a unique segment of continuous enrollment with the insurer. We allowed for small gaps up to 2 days in coverage when defining periods of continuous enrollment. We also calculated an annual rate of external turnover, defined as the share of members experiencing external turnover at least once in the calendar year. We refer to instances of external turnover as external turnover episodes, as a given member may experience external turnover multiple times if they have multiple periods of continuous enrollment with the insurer. A member observed for only 1 segment of continuous enrollment that spans the end of the study period would have no external turnover episodes. For external turnovers from group plans, we additionally separated whether the turnover was the member’s choice or the employer’s choice. We used information on the employer associated with the health plan to evaluate whether the member turnover was due to the employer discontinuing its contract with Anthem (employer choice) or whether the member disenrolled despite the employer’s contract remaining in place (member choice). In the individual market, turnover may be the member’s choice or a reflection of Anthem’s exit from select state marketplaces beginning in 2017.^20^

We defined reenrollment following each external turnover episode based on whether a subsequent enrollment segment was observed for the member after any amount of time has passed. We included returns to both commercial and noncommercial (ie, Medicare Advantage and Medicaid Managed Care, which are combined into other) lines of business. We also describe trends in reenrollment across years after the initial turnover.

### Statistical Analysis

Data analysis was conducted from January 21, 2020, through December 23, 2021. We constructed an analytic data set at the member-month level and used descriptive methods to quantify the extent of external turnover and reenrollment. We used Kaplan-Meier curves to characterize the variation in time to reenrollment following external turnover. We also identified members who experienced multiple instances of external turnover. In some analyses, we accounted for potential censoring owing to the end of the study period by limiting the sample to those whom we were able to observe for 3 years after external turnover. Because the sample of members includes both primary policyholders and dependents (eg, spouses, children) and because insurance coverage decisions may not be independent within a household, we conducted sensitivity analyses in which we separately analyzed both types of members. We also completed subgroup analyses by age (25, ≥26, 64, and ≥65 years), by health (0 vs ≥1 comorbidity), and for 4 large states where Anthem has varying market shares.

All statistical analyses were performed using Stata, version 15.0 (StataCorp LLC). Flowcharts were created using R Studio, version 4.0.2 (R Foundation for Statistical Computing).

## Results

### Sample

Our sample consisted of 3 018 633 unique members. Men (50.2%) and women (49.8%) were equally represented in the sample, and the mean (SD) age of all enrollees, including dependents, was 30.68 (19.05) years. Primary policyholders (mean [SD],  50% [0.50]) and dependents were common plan holders (eTable in the Supplement). The share of members residing in the Northeast was 18%; South, 32%; West, 23%; and Midwest, 28%, which is broadly consistent with Anthem’s presence across the US. At their first observed enrollment segment, 92% of the selected members were enrolled in the insurer’s commercial group plans and 8% were enrolled in individual plans. Most members were enrolled in preferred provider organizations (76%), with smaller shares enrolled in health maintenance organizations (14%) and consumer-directed health plans (10%).

### External Turnover

We found that 80% of Anthem members experienced external turnover during the study period. On average, each member was enrolled with the insurer for 48 months during the study period and not necessarily continuously. However, there was substantial variation in the total length of enrollment, with some members enrolled for only 1 month and others for more than 12 years (the full study period). eFigure 1 in the Supplement further characterizes the distribution of enrollment duration. About 28% of members had more than 1 enrollment segment in the study period (eTable in the Supplement).

**Figure 1.**
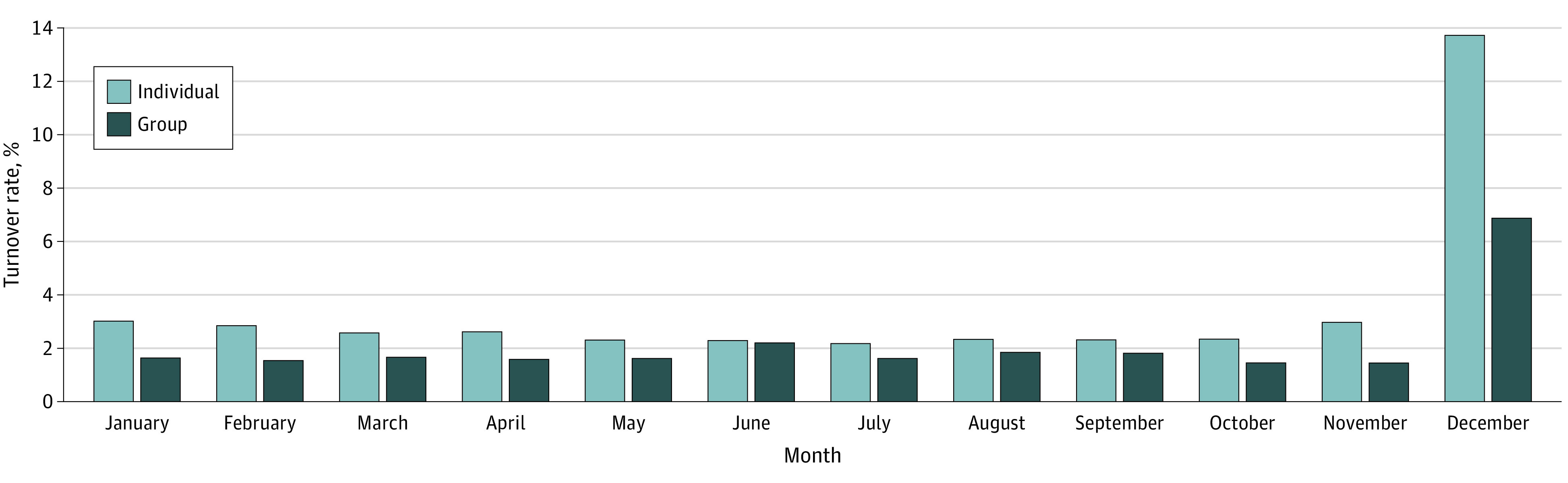
External Turnover Rates by Line of Business Monthly turnover rates by line of business across all years.

A total of 2.2% of the members experienced external turnover each month and 21.5% experienced external turnover each year. Few members experienced external turnover multiple times within a given year. Figure 1 includes the rates of external turnover by calendar month and commercial line of business (group or individual). The individual market had higher average monthly turnover (3.4%) compared with the group market (2.1%). Compared with other calendar months, December had the highest rate of external turnover: 13.8% for the individual market and 6.9% for the group market. External group turnover did not vary substantially in the state-by-state analysis for group coverage but was substantially higher in the individual market in all states (eFigure 2 in the Supplement). Among external turnovers from group plans, 25% was the result of employer choices to leave the insurer and 75% was associated with member choices to leave the insurer and/or their employer.

### Reenrollment

**Figure 2.**
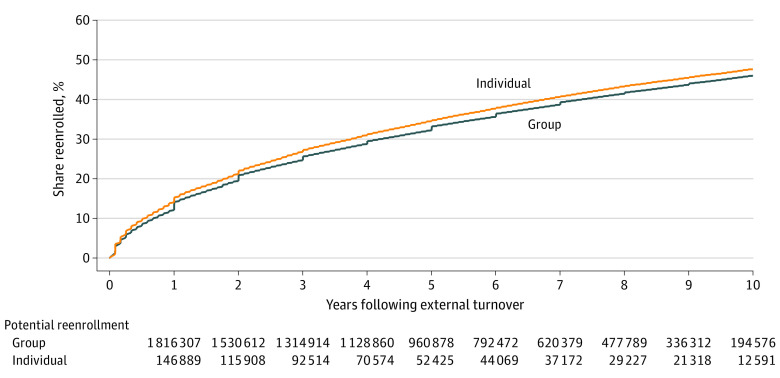
Reenrollment Following External Turnover by Line of Business at Departure Data on each member from their first episode of external turnover until reenrollment in any line of business or the end of the study period, whichever came first. By limiting the data to the first episode, the assumption that the data are independent allows the assumption that the data are independently and identically distributed across external turnover episodes, thus avoiding the possibility of serially correlated unobservable characteristics. Mortality once a member left the insurer was not documented; thus, we could not distinguish whether a former member did not reenroll due to death or choice. With this inability to account for mortality-related censoring, a lower bound on reenrollment as a share of potential returning enrollees was estimated.

We next analyzed the probability of reenrollment with the insurer following external turnover. Figure 2 displays Kaplan-Meier curves of time to first return, stratified by individual and group line of business at departure (ie, at external turnover). We found little variation in the reenrollment rates by the line of business. Specifically, 14% of the members who left the insurer from an individual plan reenrolled with the insurer after 1 year, and 34% had reenrolled after 5 years. For members who left the insurer from a group plan, 12% reenrolled after 1 year and 32% reenrolled after 5 years. After 10 years, reenrollment was 47% for both individual and group members. The eTable in the Supplement displays the socioeconomic characteristics of members who reenrolled: compared with the full sample, reenrollees were younger (mean [SD] age, 28.2 [17.2] years) and healthier (mean [SD], 0.36 [0.93] comorbidities). In addition, 22% of the members returned via the same employer.

In state-specific analyses, we observed lower reenrollment in low market-power states (28%-33%) and higher reenrollment in high market-power states (34%-43%). After 10 years, reenrollment remained higher in high market-power states (eFigure 3 in the Supplement). We also found that a substantial share of reenrollment occurred within the same state and/or line of business (>80%).

**Figure 3.**
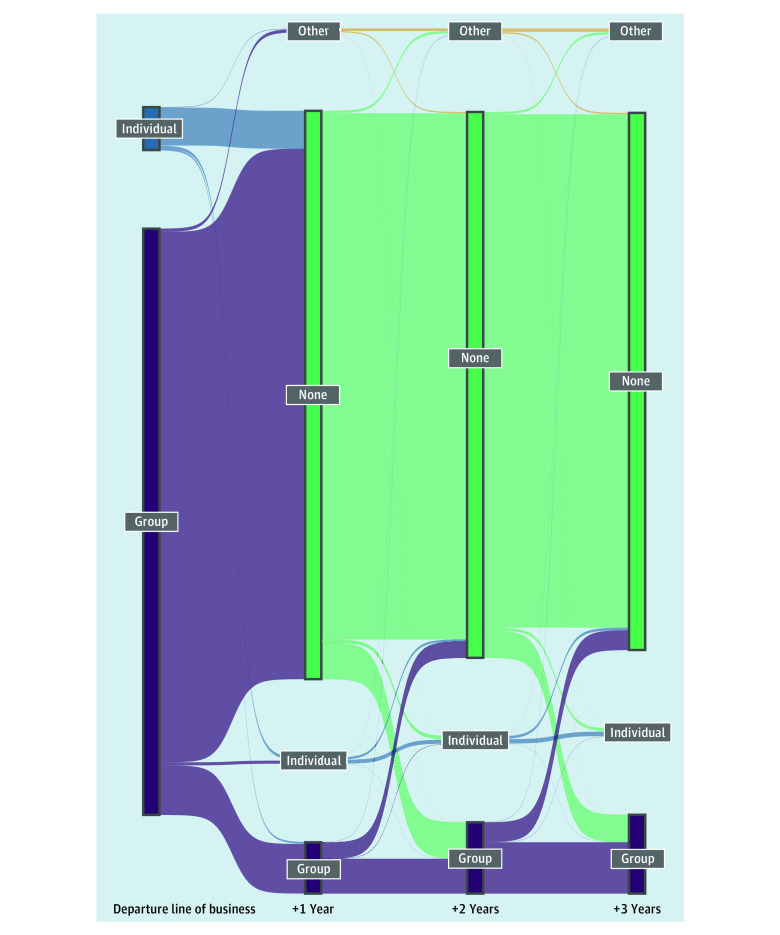
Transitions Following External Turnover (No Censoring) Members with external turnover (n = 1 887 837) that occurred 3 years before the end of the study period to avoid censoring. Each column displays the coverage type in years 1, 2, and 3 after the initial external turnover of the member.

Figure 3 depicts the flows of members in the 3 years following an external turnover among members with their first external turnover 3 or more years before the end of the study period (n = 1 887 837). eFigure 4 in the Supplement shows an analogous figure in which the sample is not restricted and censoring is an outcome (n = 2 423 297). The line of business composition of the initial external turnover episodes was 93% for group members and 7% for individual members. After 1 year, 10% of departing enrollees had returned in some capacity: 8% to the group line of business and the remainder split between individual and other. After 3 years, 15% had returned to the insurer, with most reenrolled in a group plan (86%). Although most reenrollees returned to the same line of business (81%), we also observed that 19% returned to another line of business. Figure 3 captures the subsequent episodes of external turnover following the first external turnover. This phenomenon is represented by flows from the different lines of business after 1 year to the no return outcome. Among members included in the Figure, 0.7% (n = 12 557) had more than 1 external turnover episode within the 3 years, meaning they left the insurer once, returned, and then left again.

**Figure 4.**
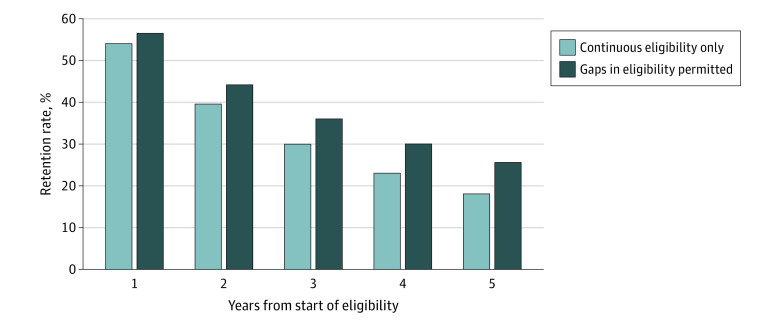
Member Retention Over Time (No Censoring) Retention rates for members in subsequent years with and without continuous eligibility from their first eligibility year-month. This share varied according to whether the member was continuously enrolled with the insurer the entire time or whether there were gaps in eligibility (ie, the possibility of return).

The member retention rate is a common business metric used by insurers when calculating the return on investment of certain health investments they could incorporate into their benefit design. In Figure 4, we show the share of members who were enrolled with the insurer at 1, 2, 3, 4, and 5 years following their first observed enrollment period. Allowing for coverage gaps raised retention rates at each year, creating a gap that widened over time. After 5 years, only 25% of the members remained continuously enrolled without any gaps; however, this share increased to 35% after accounting for members who disenrolled and subsequently reenrolled. Allowing for censoring provided similar results (eFigure 5 in the Supplement). State-specific results in eFigure 6 in the Supplement display similar continuously enrolled retention rates in the 2 low and 1 high market-power states.

### Trends Over Time in External Turnover and Reenrollment

**Figure 5.**
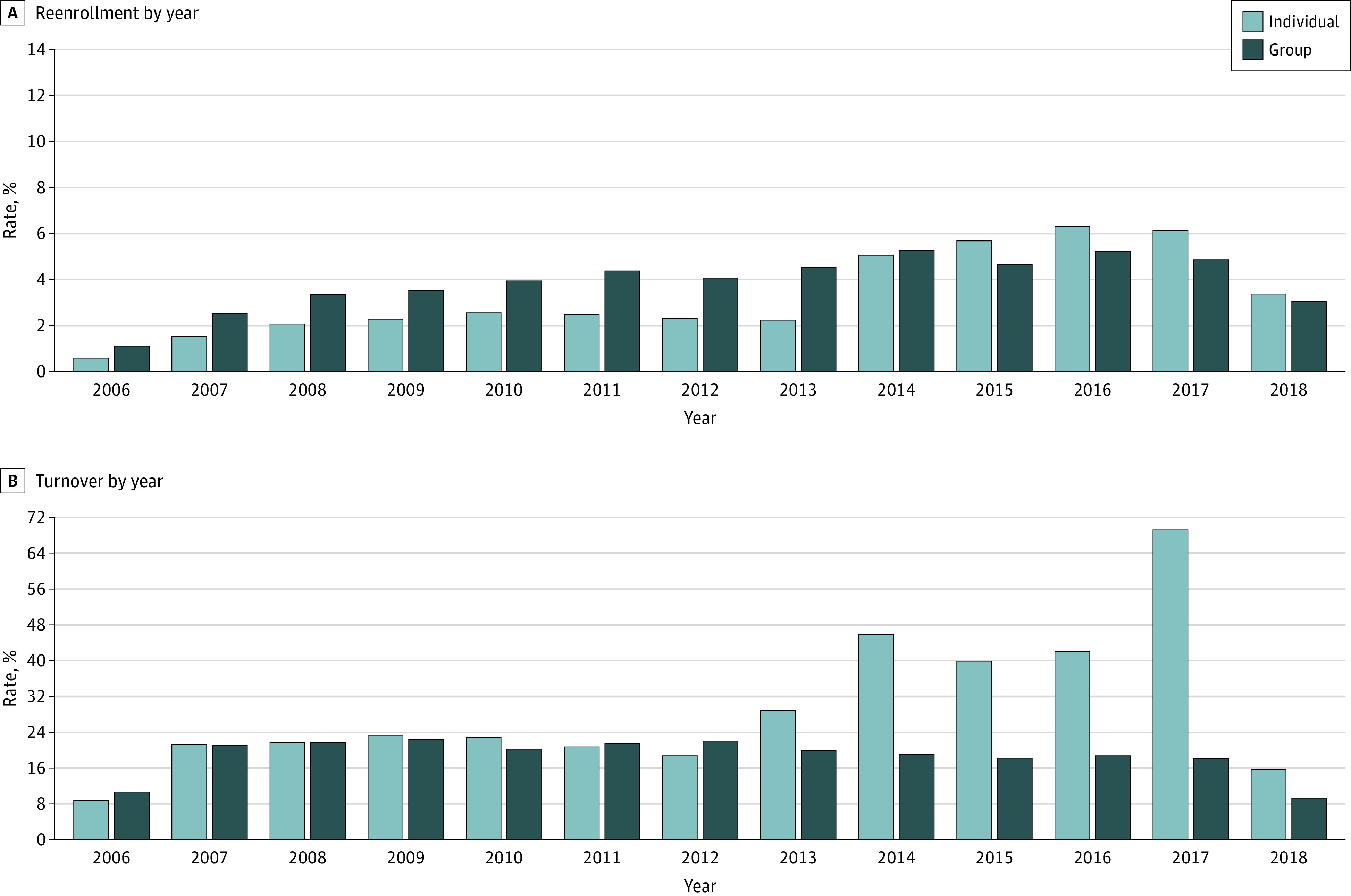
Trends in Turnover and Reenrollment Over Time Annual turnover rates and reenrollment rates by line of business across all years.

Figure 5 displays annual trends in external turnover and reenrollment by line of business. The turnover rate decreased slightly from 21% in 2007 to 18% in 2017. External turnover in the individual group market accelerated beginning in 2013 and peaked in 2017, after which Anthem stopped participating in many individual marketplaces. Reenrollment rates in the group market increased from 2007 (2.5%) to 2017 (4.5%); reenrollment rates also increased in the individual market from 1.8% in 2007 to 6% in 2017.

### Subgroup Analyses

Turnover trends did not differ from Figure 1 by primary coverage or dependent status (eFigure 7 in the Supplement). Substantially different individual turnover rates emerged by age group; adults aged 25 years had generally higher turnover in any given month (4%) and experienced higher group turnover in December (11%). Those aged 64 years experienced substantially higher turnover in the individual market with monthly rates ranging around 6% between January and November and 23% in December. Similarly high rates of individual turnover were observed for those aged 65 years or older. Turnover for healthy individuals was generally higher by approximately 1% across the individual and group markets relative to the unhealthy members.

Reenrollment rates for primary policyholders, dependents, individuals aged 25 years, those aged 26 years and older, more healthy individuals, and less healthy individuals did not vary much compared with the full sample results (eFigure 8 in the Supplement). Generally, reenrollment rates were slightly higher for the individual market than the group market and were 33% after 5 years and between 45% and 50% after 10 years. Reenrollment rates were lower for those aged 64 years and older (15% after 5 years in the individual market and 11% in the group market). After 10 years, the reenrollment rate in the group market did not surpass 20% but increased to 30% in the individual market.

## Discussion

In this study, we noted high rates of external turnover at a large national insurer. Disenrollment was common between 2006 and 2018; however, 25% of the members remained with the insurer for 5 years before disenrolling and 34% returned within 5 years after disenrolling. Accounting for such high reenrollment leads to greater retention rates, which are relevant to health investment decisions. Failure to account for reenrollment can substantially underestimate the share of members expected to be covered at a future time when health investment returns accrue.

Our finding of relatively high reenrollment likely reflects a combination of factors. The insurer’s multiple lines of business and its often large market share within these states serve to increase the probability that a given member reenrolls. However, these characteristics are not unique to Anthem. According to the Kaiser Family Foundation, the average market share for the largest insurer in each of the 50 states is above 60%.^21^ Thus, one can expect to find similar trends in reenrollment in other non-Anthem states where another insurer has the dominant market share. Our findings may also be relevant to smaller insurers that operate in more narrowly defined markets (eg, within a single region and/or line of business) but with considerable market share. Because we observed that most people in this study reenrolled in the same state and line of business, our results are unlikely to be associated with enrollees leaving the insurer because they move to another state or market.

We noted similar rates of external turnover in most months and reenrollment in Anthem’s group and individual businesses. In the commercial markets, annual open enrollment periods prevail, outside of which individuals may change their coverage only when they experience certain qualifying events, such as birth of a child or marriage. However, subgroup analyses showed higher turnover for younger individuals in the group market as well as older individuals in the individual market. Turnover differed across states, especially in the individual market, where we observed lower turnover in states where Anthem has higher market power in the individual market. Our finding that reenrollment rates were substantially higher in high market-power states suggests that market shares are important for both turnover and reenrollment.

Our findings have possible implications for current and future health policy. First, in most states, 90% of the commercial market is captured by 3 insurers or fewer.^21^ Given this level of consolidation and the increasing enrollment in Medicaid Managed Care and Medicare Advantage, concerns have been raised about insurer consolidation leading to increased market power and higher health insurance premiums.^22^ However, consolidation across and within different lines may also lead to greater continuity in insurance coverage over a person’s lifetime. Second, the shifts toward greater privatization of publicly funded insurance benefits, such as Medicaid and Medicare, will likely continue, which will further increase reenrollment rates among commercial insurers. To date, 40 states have Medicaid Managed Care contracts; enrollment grew by 20% from 2020 to 2021, and Medicare Advantage enrollment has been increasing by about 8% per year since 2010.^23^ Federal policy discussions, such as Medicare-for-All, which may entail expansions through Medicare Advantage plans, would lead to additional growth in publicly funded insurance benefits administered through commercial insurance companies. Overall, the current health insurance policy sentiment favors growing enrollment and reenrollment among commercial insurance companies across all lines of business.

In addition, our findings carry implications for insurance benefit design, particularly in regard to the extent of mandated care benefits. Certain benefits, such as a policy of no cost-sharing for diabetes care, may be more or less important to mandate depending on how much reenrollment might mitigate underinvestment in these benefits. An insurer must weigh upfront costs of preventive care, screenings, and treatments against the likelihood that any future cost-savings will be realized while the member is still enrolled. High turnover and greater time between the investment and its future payoff will discourage the coverage of a given service. However, our findings suggest that the probability of reenrollment should also be factored into this decision. When upfront investment costs are low relative to eventual cost-savings, an insurer may find it in their best interest to cover a service at an earlier time so long as a sufficiently large share of members are expected to be covered at the time when savings will be realized. A higher rate of reenrollment upon disenrollment may reduce the leakage of the cost-saving benefits of preventive care investments by the insurer, thus better incentivizing the insurer to offer such benefits.

### Limitations

This study has limitations. First, our analysis was limited to one insurer that operates plans across the country. Although our findings may be generalized to other for-profit and nonprofit insurers, they may not generalize to smaller insurers with operations limited to a single line of business. However, even regional insurers often command a large market share in their respective areas.^21^ Second, our data are subject to censoring. We did not observe the duration of tenure with the insurer for members enrolled in the first month of the study period. Similarly, right-censoring limited our ability to observe reenrollment that occurred beyond the end of the data window. Third, we may have underestimated the rates of reenrollment among living persons, particularly older members in our sample because we did not have mortality data.

## Conclusions

In this study, we observed that the commercial health insurance market displays a substantial level of external turnover. However, many individuals also reenrolled with the same insurer within 5 years. The findings imply that it may be useful for insurers to focus on the long-term health of individuals because many members will return to the same insurer within a relatively short period.

## Notes

Supplement. **eMethods.** Data Linkage and Sample Construction **eTable.** Member Characteristics of Full Sample and Reenrollees **eFigure 1.** Distribution of the Length of Total Enrollment and Continuous Enrollment Segments **eFigure 2.** External Turnover Rates by Line of Business in States With Low and High Insurer Market Power **eFigure 3.** Reenrollment Following External Turnover by Line of Business at Departure in States with Low and High Insurer Market Power **eFigure 4.** Transitions Following External Turnover (With Censoring) **eFigure 5.** Member Retention Over Time (With Censoring) **eFigure 6.** Member Retention Over Time (Without Censoring) in States with Low and High Insurer Market Power **eFigure 7.** External Turnover Rates by Line of Business by Primary Coverage, Age, and Health **eFigure 8.** Reenrollment Following External Turnover by Line of Business by Primary Coverage, Age, and Health
