# Supplementary material for: Trends in Disenrollment and Reenrollment Within US Commercial Health Insurance Plans, 2006-2018
Source: JAMA Netw Open. 2022 Feb 24;5(2):e220320. doi: 10.1001/jamanetworkopen.2022.0320 (PMC8874349; doi:10.1001/jamanetworkopen.2022.0320)
Supplement: Supplement. — eMethods. Data Linkage and Sample Construction eTable. Member Characteristics of Full Sample and Reenrollees eFigure 1. Distribution of the Length of Total Enrollment and Continuous Enrollment Segments eFigure 2. External Turnover Rates by Line of Business in States With Low and High Insurer Market Power eFigure 3. Reenrollment Following External Turnover by Line of Business at Departure in States with Low and High Insurer Market Power eFigure 4. Transitions Following External Turnover (With Censoring) eFigure 5. Member Retention Over Time (With Censoring) eFigure 6. Member Retention Over Time (Without Censoring) in States with Low and High Insurer Market Power eFigure 7. External Turnover Rates by Line of Business by Primary Coverage, Age, and Health eFigure 8. Reenrollment Following External Turnover by Line of Business by Primary Coverage, Age, and Health [file jamanetwopen-e220320-s001.pdf]

Supplementary Online Content

Fang H, Frean M, Sylwestrzak G, Ukert B. Trends in disenrollment and reenrollment within US commercial health insurance plans, 2006-2018. *JAMA Netw Open*. 2022;5(2):e220320. doi:10.1001/jamanetworkopen.2022.0320

eMethods. Data Linkage and Sample Construction

eTable. Member Characteristics of Full Sample and Reenrollees

eFigure 1. Distribution of the Length of Total Enrollment and Continuous Enrollment Segments

eFigure 2. External Turnover Rates by Line of Business in States With Low and High Insurer Market Power

eFigure 3. Reenrollment Following External Turnover by Line of Business at Departure in States with Low and High Insurer Market Power

eFigure 4. Transitions Following External Turnover (With Censoring)

eFigure 5. Member Retention Over Time (With Censoring)

eFigure 6. Member Retention Over Time (Without Censoring) in States with Low and High Insurer Market Power

eFigure 7. External Turnover Rates by Line of Business by Primary Coverage, Age, and Health

eFigure 8. Reenrollment Following External Turnover by Line of Business by Primary Coverage, Age, and Health

This supplementary material has been provided by the authors to give readers additional information about their work.

© 2022 Fang H et al. *JAMA Network Open.*

eMethods. Data Linkage and Sample Construction

Data Linkage:

The HIRD is able to connect eligibility segments over time through an algorithm that was established over 9 years ago and quality checked. The algorithm relies on information on name, SSN, age, gender, addresses, and proprietary Anthem member identifiers (such as Subscriber ID, Member Keys) to connect eligibility segments for the same person across time.

Sample Construction:

We use data from a 5% random sample of commercially covered members drawn between 2006 and August 2018. For each member selected in the sample we pull in all eligibility segments, including coverage from commercial coverage and other lines of business. This provides us with a full census of all coverage between 2006 and 2018 for the 5% sample.

© 2022 Fang H et al. *JAMA Network Open.*

eTable: Member Characteristics of Full Sample and Reenrollees

		Full Sample			Returners	
Characteristic	Total	Group	Individual	Total	Group	Individual
N	3,018,633	2,789,392	217,931	831,710	768,858	62,852
Age	30.68	30.5	33.04	28.20	28.08	29.72
Female	49.80%	49.60%	52.10%	49.85%	49.65%	52.24%
Primary Subscriber	50.00%	49.20%	63.30%	49.14%	48.14%	61.41%
Elixhauser Index	0.59	0.61	0.41	0.36	0.37	0.26
Region						
Northeast	17.60%	18.50%	6.40%	15.87%	16.87%	4.59%
South	31.90%	32.30%	26.70%	33.09%	33.25%	31.33%
Midwest	22.90%	24.20%	8.60%	23.64%	25.25%	5.44%
West	27.60%	25.00%	58.30%	27.40%	24.63%	58.65%
Product type						
HMO	13.10%	12.40%	21.80%	16.44%	16.52%	15.44%
PPO	76.90%	77.20%	72.90%	77.51%	77.27%	80.41%
CHDP	10.10%	10.40%	5.30%	6.06%	6.21%	4.16%
Any Turnover	88.10%	87.50%	96.00%	100%	100%	100%
Any External Turnover	80.30%	79.40%	91.80%	100%	100%	100%
Share who return to Insurer	34.80%	34.90%	32.80%	100%	100%	100%
Segments of Continuous Enrollment						
Mean	1.39	1.39	1.41	2.35	2.35	2.32
SD	1.16	0.78	0.74	0.88	0.88	0.73
Range	1 – 41	1 – 41	1 – 22	2 – 41	2 – 41	2 – 22
Share with >1 segment	27.90%	27.80%	30.10%	100%	100%	100%
Return to same employer					22.08%	
Total Months Observed						
Mean	48.77	49.38	40.81	58.42	58.89	52.48
SD	40.82	40.85	39.69	36.17	36.08	36.87
Range	1 - 152	1 – 152	1 – 152	2 – 152	2 – 152	2 – 152
Left-censored (%)	1.40%	1.40%	1.90%	3.00%	3.54%	2.53%

Notes Mean sample averages of demographic and plan characteristics. HMO = health maintenance organization; PPO = preferred provider organization; CDHP = consumer-directed health plan; SD = standard deviation. Member Characteristics are displayed based on their first month enrollment segment with the exception of the Elixhauser Index which is a count of 31 possible Elixhauser comorbidities diagnosed within the 12 months prior to the individual's end of the first enrollment segment.

© 2022 Fang H et al. *JAMA Network Open.*

eFigure 1: Distribution of the Length of Total Enrollment and Continuous Enrollment Segments

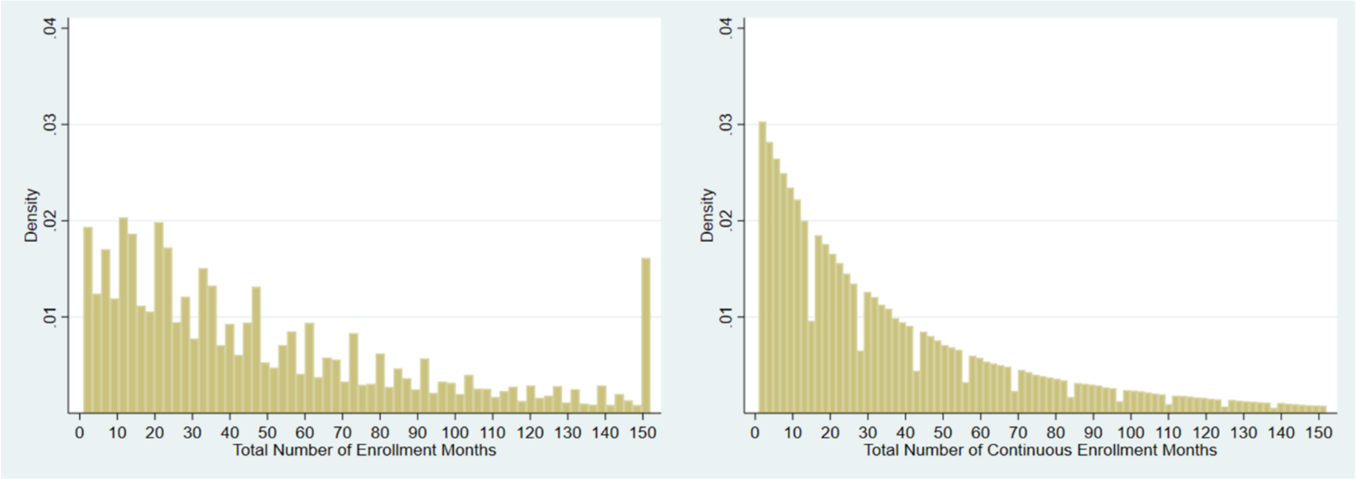

Notes Left figure displays the distribution of the total length of all eligibility segments for a member, while the right figure displays the length of each continuous eligibility segment.

© 2022 Fang H et al. *JAMA Network Open.*

eFigure 2: External Turnover Rates by Line of Business in States with Low and High Insurer Market Power

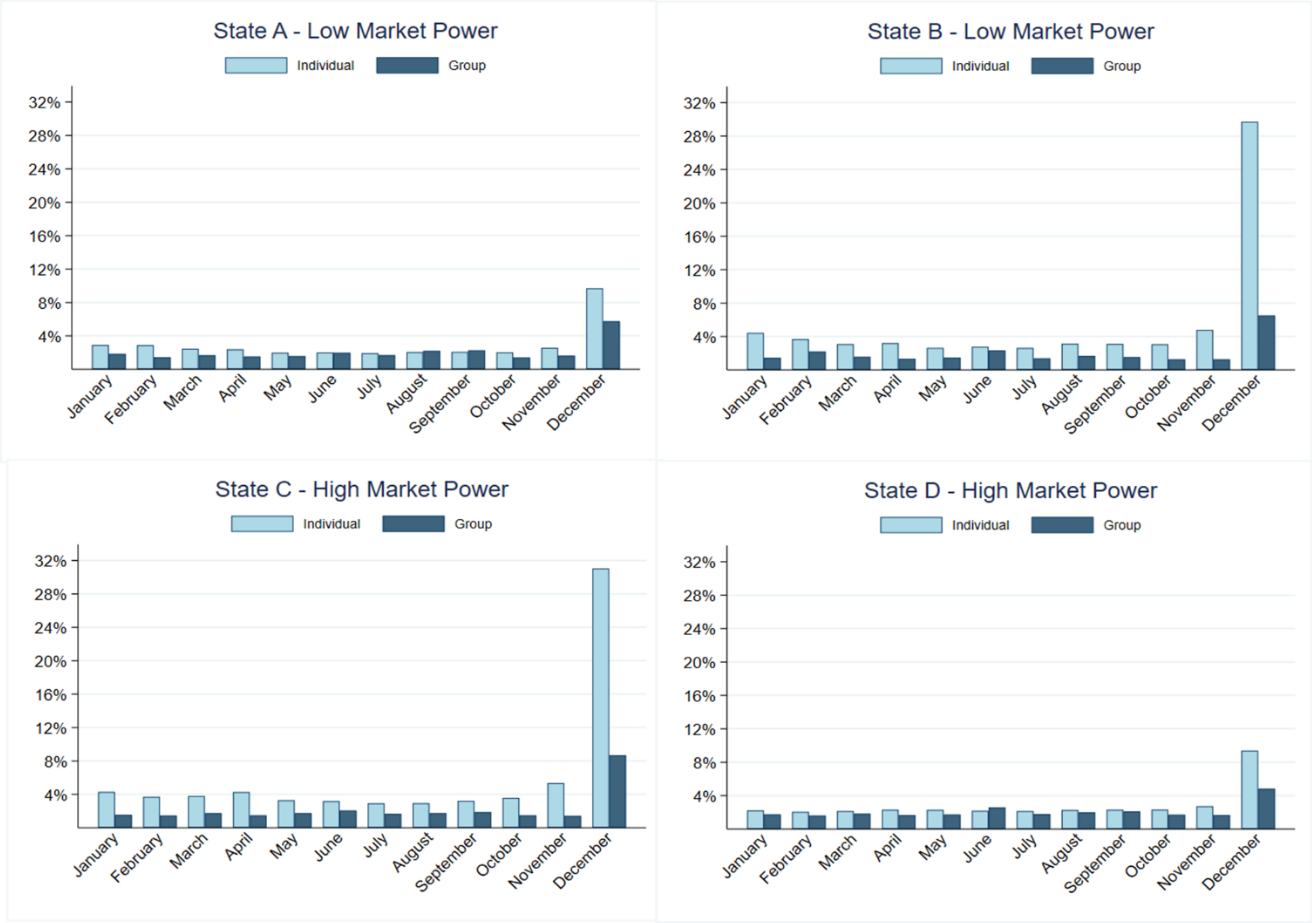

Notes Monthly turnover rates by line of business across all years by state market power. Market power is defined at the state level based on all members covered in the commercial market (including the individual market) relative to the size of the commercial market in a state. High market power states are those with a market share of more than 40% and low market share states are defined as having a share of less than 30%. Market shares only based on the individual market shows that State A and D had high market power in the individual market (above 45%).

© 2022 Fang H et al. *JAMA Network Open.*

eFigure 3: Reenrollment Following External Turnover by Line of Business at Departure in States with Low and High Insurer Market Power

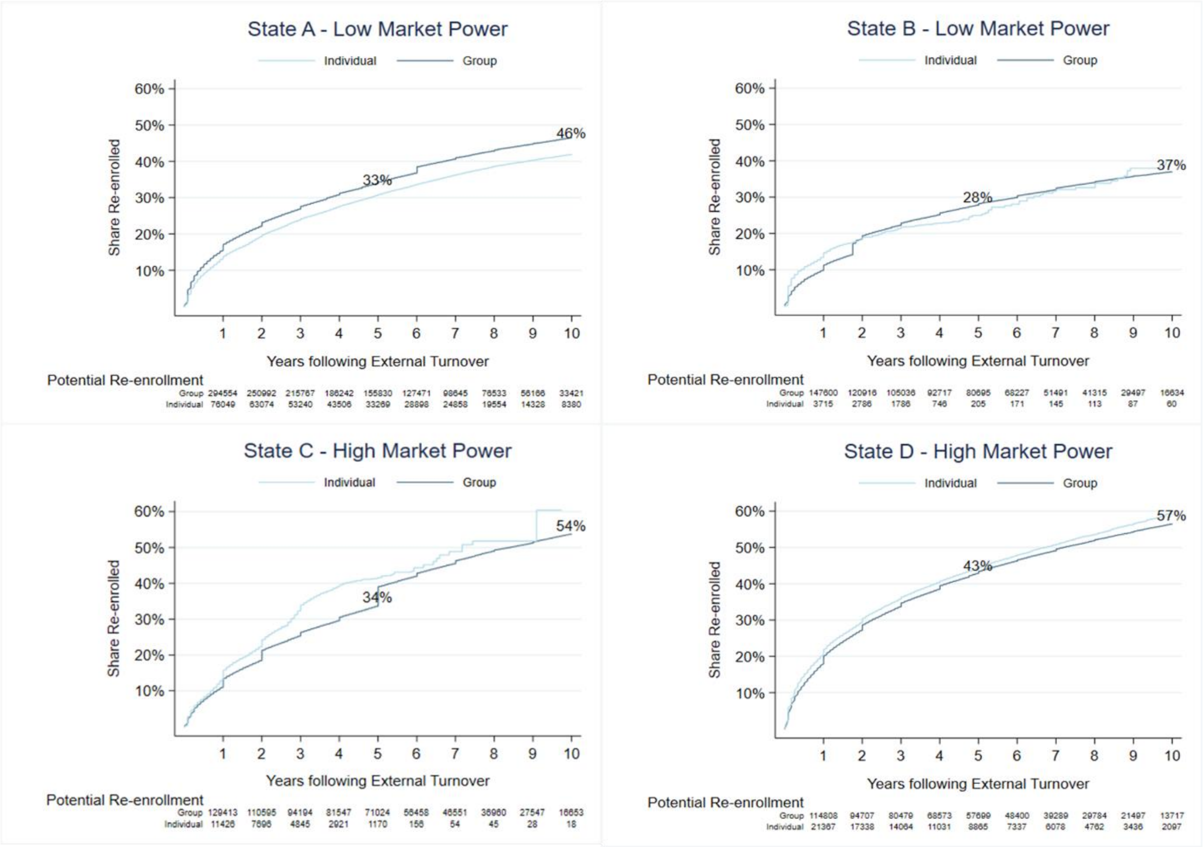

Notes Kaplan Meier Survival Curves by line of business measuring the time until a member re-enrolls with the insurer measured from the time the member left. Market power is defined at the state level based on all members covered in the commercial market relative to the size of the commercial market in a state. High market power states are those with a market share of more than 40% and low market share is defined as having a share of less than 30%. Market shares only based on the individual market shows that State A and D had high market power in the individual market (above 45%)

.

© 2022 Fang H et al. *JAMA Network Open.*

eFigure 4: Transitions Following External Turnover (with censoring)

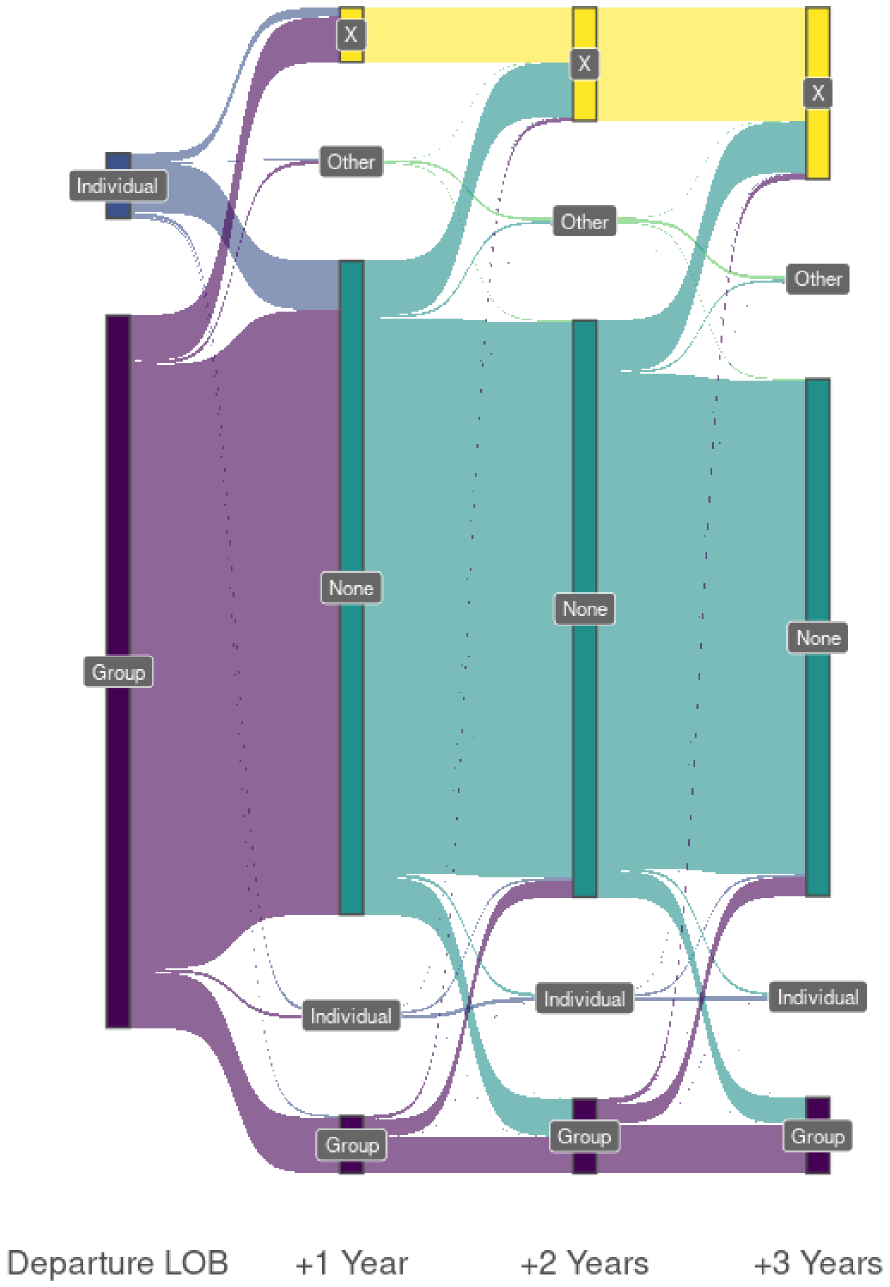

Notes Table displays only members with external turnover (N=2,423,297). Each column displays the coverage type in year 1, 2, and 3 after the initial external turnover of the member with potential censoring. X displays that this member was not observed in the data anymore.

© 2022 Fang H et al. *JAMA Network Open.*

eFigure 5: Member Retention Over Time (with censoring)

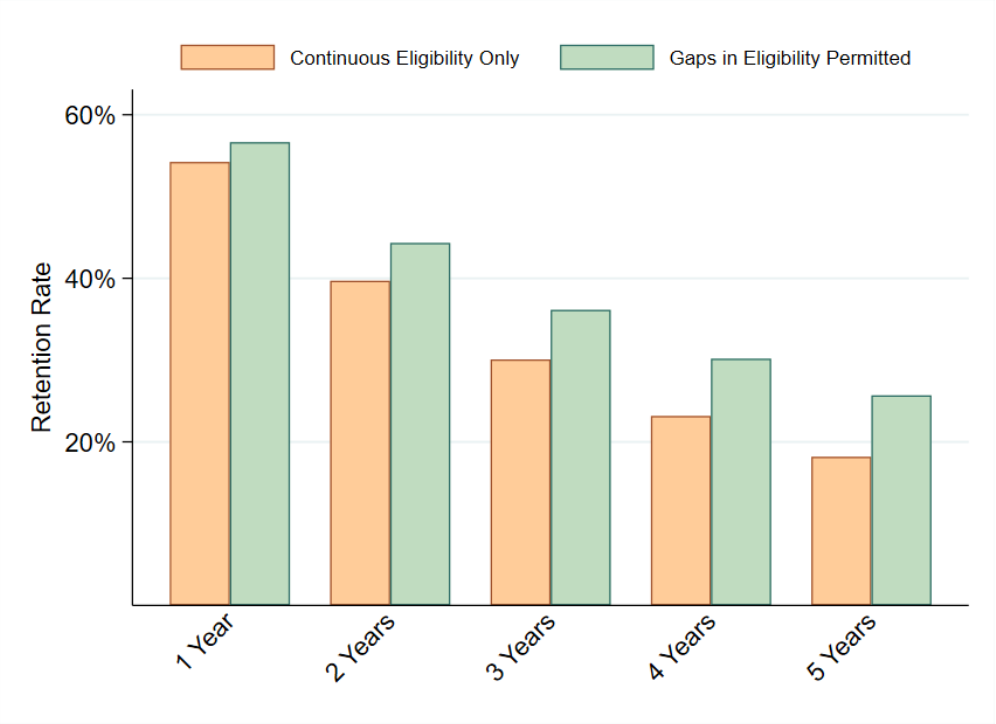

Notes Retention rates allowing for censoring for members in subsequent years with and without continuous eligibility from their first eligibility year-month.

© 2022 Fang H et al. *JAMA Network Open.*

eFigure 6: Member Retention Over Time (without censoring) in States with Low and High Insurer Market Power

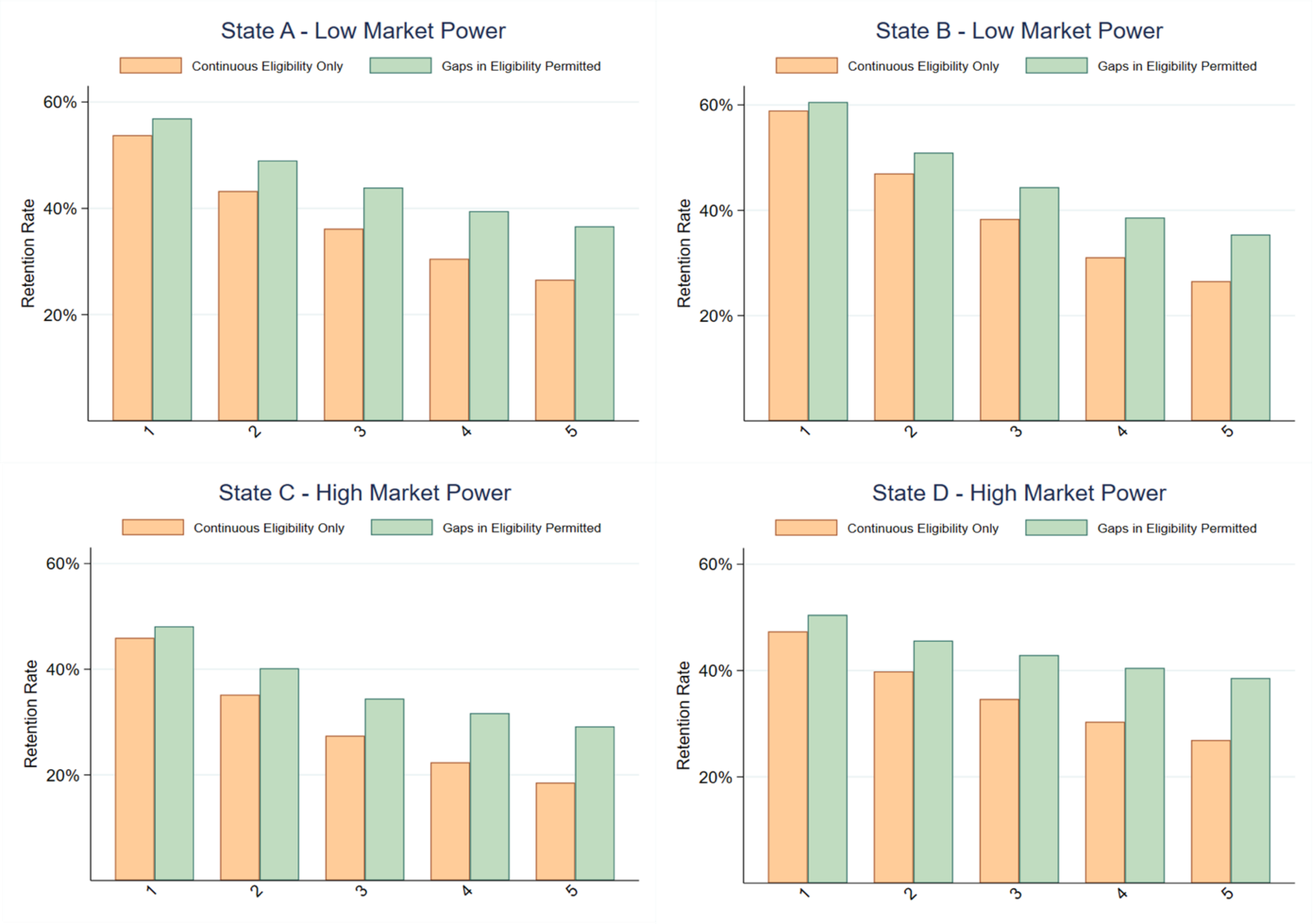

Notes Retention rates for members in subsequent years with and without continuous eligibility from their first eligibility year-month. Market power is defined at the state level based on all members covered in the commercial market relative to the size of the commercial market in a state. High market power states are those with a market share of more than 40% and low market share is defined as having a share of less than 30%.

© 2022 Fang H et al. *JAMA Network Open.*

eFigure 7: External Turnover Rates by Line of Business by Primary Coverage, Age, and Health

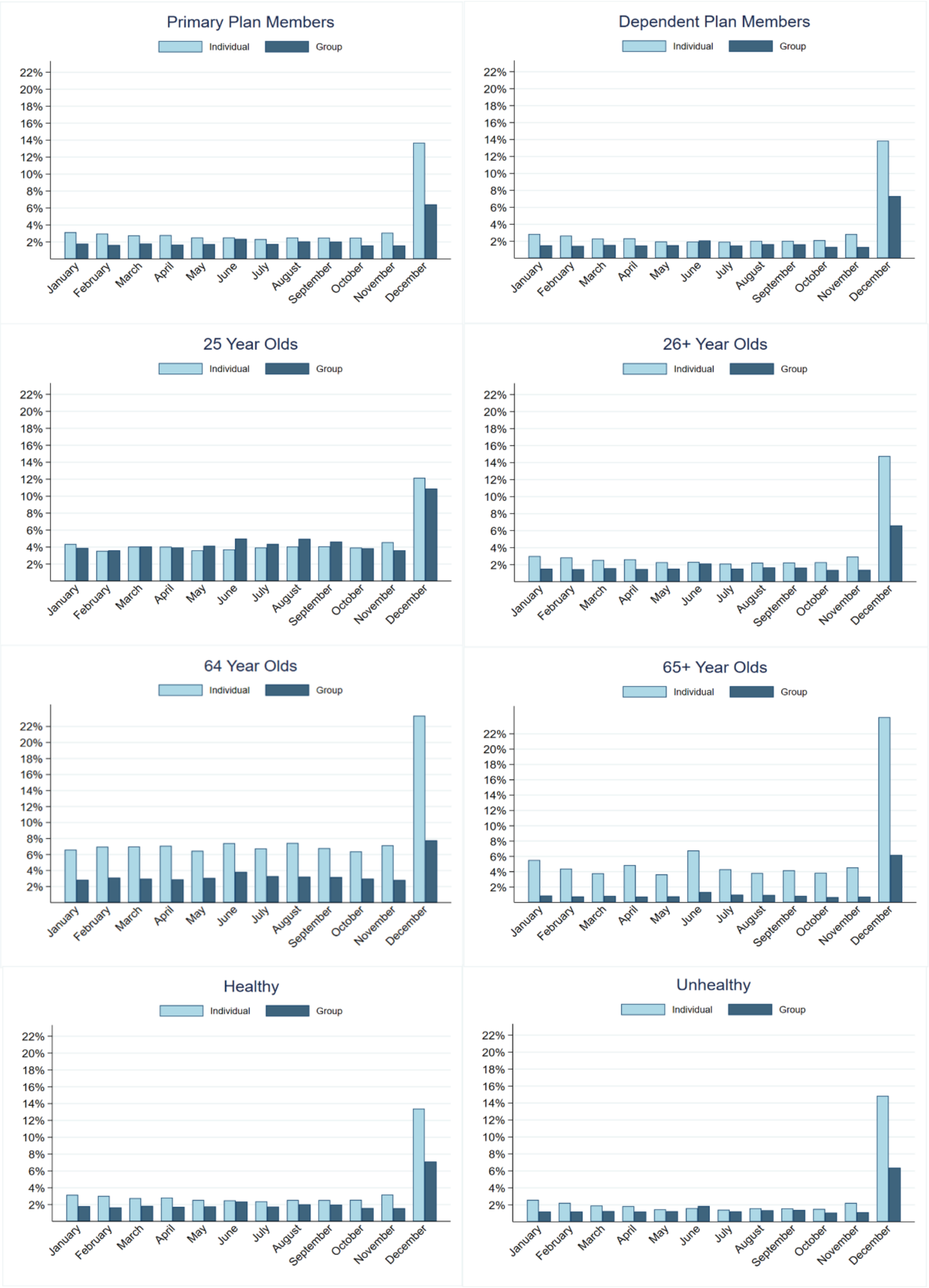

Notes Monthly turnover rates by line of business across all years for select groups of individuals. Healthy members are defined as those with no Elixhauser comorbidities, and unhealthy members are those with one or more Elixhauser comorbidities.

© 2022 Fang H et al. *JAMA Network Open.*

eFigure 8: Reenrollment Following External Turnover by Line of Business by Primary Coverage, Age, and Health

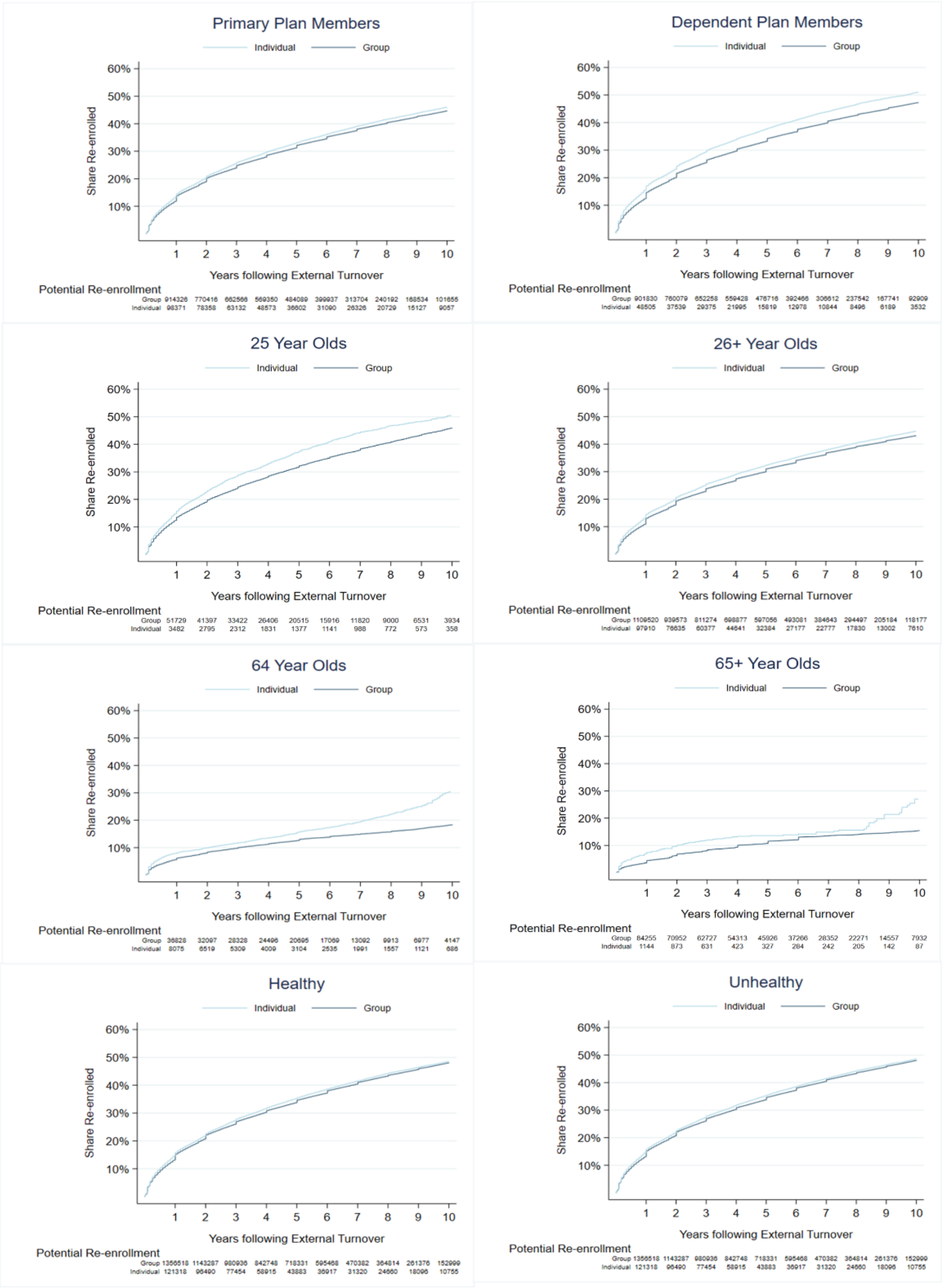

Notes Kaplan Meier Survival Curves by line of business measuring the time until a member re-enrolls with the insurer measured from the time the member left for select groups of individuals. Healthy members are defined as those with no Elixhauser comorbidities, and unhealthy members are those with one or more Elixhauser comorbidities.

© 2022 Fang H et al. *JAMA Network Open.*
